# Promoting Mental Health in Italian Middle and High School: A Pilot Study

**DOI:** 10.1155/2017/2546862

**Published:** 2017-05-31

**Authors:** Franco Veltro, Valentina Ialenti, Manuel Alejandro Morales García, Emiliana Bonanni, Claudia Iannone, Marinella D'Innocenzo, Antonella Gigantesco

**Affiliations:** ^1^Dipartimento di Salute Mentale di Campobasso, ASReM, Campobasso, Italy; ^2^Regione Molise, Campobasso, Italy; ^3^Istituto Superiore di Sanità, Roma, Italy

## Abstract

**Aim:**

In Italy, a handbook has been developed based on the principles of cooperative learning, life skills, self-effectiveness, and problem-solving at high school level. Early studies have shown the handbook's effectiveness. It has been hypothesized that the revised handbook could be more effective in middle schools.

**Method:**

The study design is a “pre- and posttest” that compares the results obtained from 91 students of the high schools with those of the 38 students from middle schools. The assessment was made through “self-reporting” questionnaires of (a) learning skills including problem-solving and (b) perceived self-efficacy in managing emotions, dysfunctional beliefs, and unhealthy behaviours (i.e., drinking/smoking).

**Results:**

Significant improvements were observed in both groups with the exceptions of perceived self-efficacy in managing emotions. The improvement of dysfunctional beliefs and the learning of problem-solving skills were better in middle schools.

**Conclusion:**

The results confirm the authors' hypothesis that the use of this approach is much more promising in middle school.

## 1. Introduction 

 Adolescence is the most important period for laying the foundations for psychological well-being. The estimated prevalence of worldwide mental health problems among young people associated with school failure, delinquency, and substance misuse is 10–20% [1, 2]. Promoting positive mental health may provide young people with the necessary life skills and resources to accomplish their potential and to deal with adversity [3]. School is one of the most important contexts for this initiative [3, 4] especially if the health promotion programmes are those undertaken as part of school activities that improve social and life skills [5–7]. Life skills [7, 8] are psychosocial competencies that help people to be more aware in the process of decision-making, solving problems, thinking critically and creatively, communicating effectively, developing safe relationships, understanding the emotions of others, and managing their lives in a healthy and productive manner; the most important are self-efficacy, problem-solving, empathy, and coping strategies [9].

An effective programme for promoting mental health includes sequenced step-by-step training; active forms of learning; focus on skills development; explicit learning goals (the SAFE approach) [8]. Furthermore, sequenced training especially for life skills [10] “will not be as effective unless active forms of learning are used” with “sufficient time focused on reaching explicit learning goals” [11] according to the theory of the Social Skill Training, the most effective intervention to acquire skills in psychiatry [12]. Also in educational science, skills need to be fragmented into smaller steps in order to be mastered. For this purpose lesson plans written in a handbook are very useful with suggestions of practices that are necessary for skills acquisition [13, 14].

Finally, an effective programme needs a handbook where the work is sequenced in units; for each unit a sequence of steps should be described. Reading and discussing the handbook as a group is a form of active and cooperative learning. In addition, role-play and exercises linked to the steps are necessary and should be clearly described and solicited for the reinforcement of skills.

On the basis of this premise, the Italian National Institute of Health developed a structured handbook as part of the “Gaining Health” project (http://www.ccm-network.it) in order to promote mental health among students attending high schools. It was designed to reach all students, regardless of their level of risk in developing mental/behavioural problems, in order to promote self-efficacy, psychological well-being, and satisfaction with life [15]. The handbook combined training regarding problem-solving and the development of emotional intelligence skills. It was inspired by Goleman's five domains of emotional intelligence model [16] and by Falloon's psychoeducational approach [17] whereas psychiatric patients and their families are trained to use structured problem-solving to address problems that cause them the most stressful situations in their life and to use their social network to obtain the support of the people who are most willing and able to assist them in resolving problems. As in Falloon's approach, the core component of the handbook is defining personal goals [17–19].

Two studies were conducted to evaluate the effectiveness of handbook's implementation [5, 20]. The results were positive but were not considered by the authors as satisfactory as they expected; they found it was necessary to include a notebook to stimulate homework that had been neglected by the students, allowing students to assiduously apply and consolidate skills outside the classroom. They also modified some units and simplified a lot the language. As a consequence, in a third effectiveness study further improvements were found [21].

After these positive results, the authors hypothesized applying the modified handbook in the middle school for young pupil less than 15 according to the Social Learning Theory of Bandura [22] and WHO [7] that consider some competences related to psychological well-being more easily modified at younger ages. In fact, in the school setting, late childhood and early adolescence are critical moments of opportunity for building skills and positive habits [7].

The present study aims to evaluate the effectiveness of the modified handbook, both among students 12–14 years of age attending middle school classes and students 15-16 years of age attending high school.

We hypothesized that the handbook would have brought out some differences between middle school and high school students in favour of middle school because competences regarding life skills are more sensitive to being modified in an earlier stage of development.

## 2. Method

### 2.1. The Original and Modified Version of the Handbook

 The main contents of the two handbooks (Table 1) concern a structured* six-step *problem-solving approach, defining personal goals, adopting effective communication skills, using negotiation, coping with stress and anger, resolving conflict, and recognizing and modifying negative dysfunctional beliefs that precede, accompany, and follow unpleasant emotions. The handbook mainly consists of exercises to be conducted in school and at home [23].

For Each work-unit the sequenced steps are definition of the content; emotional roll call; To verify the homework was assigned among 2-3 students randomly, with a focus on their personal goals; 3-4 students in turn read the content of the unit and the instructions to perform exercises (2-3 for each unit) in a small group linked to the content, followed by feedback, homework assignment.

A modified version of the handbook was designed to render it as “user friendly” as possible for middle school. Some units were split, some were abolished (Table 1), the language was simplified, and more exercises were added with 60% of total time dedicate to role-playing. A specific homework notebook was developed.

### 2.2. Study Design

For each group (middle school and high school classes) a “pre-post test” design study was used comparing self-administered instruments scores obtained before and after implementation of the handbook's following topics: (a) learning skills (i.e., defining goals, communication skills, and problem-solving); (b) regulating emotions; (c) having functional thoughts; (d) having healthy behaviours.

A comparison between the two groups about the same topics was made before and after the handbook's implementation.

**Figure 1:**
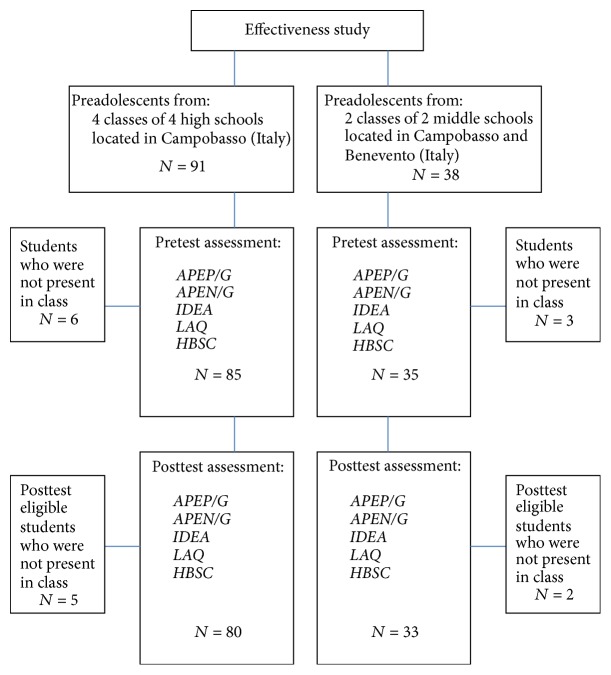
Participants flow chart.

The participating schools were contacted in early 2011 and the programme was illustrated to headmasters and teachers in each school. Three teachers of 4 classes in 4 high schools and 2 classes in two middle schools located in two cities in Southern Italy volunteered to participate in the study and put into action the implementation of the handbook as part of their curricular activities. The students' socioeconomic level was comparable in all classes; that is, the students came from the middle social class and were native Italian. Finally, the implementation of the handbook was conducted during the 2011-12 school year. These classes were composed of 91 high school students and 38 middle school students. 11 high school students and 5 middle school students were not present in class when the pretest and/or the posttest assessment/s was/were administered, or presented incomplete questionnaires (Figure 1).

### 2.3. Implementation of the Handbook

The implementation was held during regular school hours and each unit of the handbook required a one-hour session of work a week. Therefore 20 sessions were held for six months.

### 2.4. Facilitators

Each session was coordinated by a facilitator, psychologist/pedagogist, that, after reading the handbook, completed the training through a one-day session conducted by the handbook's authors; they also received a guide with practical information. It is worth noting, however, that the handbook was geared towards the students themselves, as opposed to teachers or facilitators, stressing that the students were the real protagonists and the role of the facilitator was just to stimulate the active participation of all students in class and to ensure that sessions were conducted as described in the handbook.

The facilitators did not need a specific professional specialisation (e.g., behavioural or psychodynamic for psychologists, or a degree in philosophy rather than mathematics for teacher/pedagogist) because they are facilitators and they needed just basic skills to manage groups. Incidentally they were psychologists with a training in behavioural-cognitive therapy. In addition, teachers did not have active part in the running of the programme and they were present just to support the programme with their physical presence in order to back the importance of the approach. 

Before the study began, the facilitators held two meetings with the headmasters and teachers to discuss the handbook and its objectives and met parents to obtain their informed consent.

### 2.5. Self-Administered Instruments

#### 2.5.1. Perceived Self-Efficacy Scale [24] for the Management of Positive (APEP/G) and Negative Emotions (APEN/G)

It includes the subscale on positive emotions (7 items) that refers to one's ability to experience positive emotions, such as happiness, enthusiasm, tenderness, affection, and contentment; the subscale on negative emotions (8 items) that refers to one's ability to improve negative emotional states once they are aroused in response to adversity or frustrating events. For each item, students rated, ranging from 1 (not well at all) to 5 (very well), their ability to manage their emotional states. The minimum score for the subscale of positive emotions (APEP/G) is 7 and the maximum is 35, while for the subscale negative emotions (APEN/G) they are, respectively, 8 and 40. Higher scores mean greater ability to manage emotions.

In the present study this instrument has shown good internal consistency reliability (Cronbach's alpha = 0.83).

#### 2.5.2. Inventory Idea Questionnaire [25]

It consists of 19 items related to irrational and dysfunctional beliefs. This is the relevant tool to evaluate the contents of Work-Units 9 and 10 (Table 1). An example item is “*I feel stupid if I cannot be as successful as others*” with four possible responses (strongly agree, somewhat agree, somewhat disagree, and strongly disagree). The items of the Idea Inventory are presented as an irrational idea, and any agreement represents irrational thinking. For each item, students rated, ranging from 1 (strongly agree) to 4 (strongly disagree). The minimum score is 19 (the greatest irrational beliefs) and the maximum score is 76 (the greatest functional beliefs).

In the present study the instrument has shown good internal consistency (Cronbach's alpha = 0.84).

#### 2.5.3. Learning Abilities Questionnaire (LAQ)

This tool, elaborated ad hoc, evaluates the learning (as reported by the students themselves) of 6 skills through 6 items, one for each skill: item 1 for goal Definition (Work-Units 3 and 4); items 2–5 for the communication skills (Work-Units 5–8); item 6 for problem-solving (Work-Unit 18).

The item “1” evaluates the acquisition of the characteristics of the SMART-goal (Specific, Measurable, Action-oriented, Realistic, and Time-based). For each SMART characteristic used by the student to describe his goal a score of 1 is assigned. As a result the score ranges from 0 to 5.

The items “2–5” are related to the four communication skills. Each communication skill is composed of 4 components. The score is 1 for each component mentioned; otherwise it is 0. As a consequence for each item the score ranges from 0 to 4.

The item “6” evaluates the knowledge of the six steps of problem-solving. For each step mentioned the score is 1. As a consequence the score ranges from 0 (no steps are known) to 6 (all steps are known).

The Total Score of the “LAQ” ranges from 0 (no knowledge at all) to 27 (5 points for the first item plus 16 points for the four items of communication skills plus 6 points for the item of problem-solving).

The internal consistency coefficient of this questionnaire was not calculated because it is not relevant in this case. In fact, the items of this questionnaire were designed to measure the learning process and not to measure consistent dimensions that contribute to the assessment of a unitary psychological or cognitive construct.

#### 2.5.4. HBSC for Health Behaviours

Health behaviours were assessed through self-reported items developed within the Italian HBSC-Study [26, 27]. In the present study we were interested in smoking, alcohol use, social relationships, and overall quality of life. These items were already tested in previous Italian HBSC-Surveys [28]. Smoking is assessed by asking students about the number of cigarettes smoked in the last 30 days. Alcohol consumption is assessed by asking students if they had consumed six or more alcoholic drinks on a single occasion in the last 30 days (yes; no) and if they had got drunk in the last 30 days (never; once; 2-3 times, 4–10 times, more than 10 times). Social relationships are assessed by asking about the average number of close male/female friends. Subjective quality of life is assessed by asking students to rate their present life on a visual analogue 0–10 scale with three anchor points (0 = horrible life; 6 = so and so; 10 = wonderful, the best possible life).

### 2.6. Statistical Analysis

As regards the scores, given that the assumptions of parametric statistics were not satisfied because the sample size was small, nonparametric tests were used. The Wilcoxon signed-rank test was used to compare scores obtained in the pre- and postimplementation for each group. The Mann–Whitney  *U* Test was used to compare scores between groups at pre- and postimplementation.

All analyses were performed using SPSS 19.0 (SPSS Inc., Chicago, USA) for Windows (IBM).

## 3. Results

### 3.1. Sample

A total of 113 students (51% males) completed both pre- and posttest. The age range of the sample was 12–17 years-old, with a median of 14.8 (SD = 1.4) in the initial sample and 15.1 (SD = 1.4) at the end of the handbook's implementation. Immigrants were not present; there were only 2 foreigners, who had been living in Italy for more than 8 years. The percentage of divorced parents was 12%, equally distributed in the classes. There was socioeconomic homogeneity in the sample.

### 3.2. High School Pre-Post Test: Differences (Table 2)

*APEP/G (Positive Emotions)*. No significant difference was found (difference = 0.3; *p* = 0.329).

*APEN/G (Negative Emotions)*. A significant improvement was observed (mean-ranks difference = 7.3, *p* = 0.012).

*Idea Inventory Questionnaire*. We did not find a statistically significant improvement in the overall score.

*LAQ*. We found significant improvements in the overall score (difference = 17.5; *p* < 0.01), in the* goal definition* (mean-ranks difference = 6.78; *p* < 0.01), in* expressing positive feelings* (difference = 7.2; *p* < 0.01), in* making requests *(difference = 2.86; *p* < 0.05), and in* expressing unpleasant feelings* (difference = 7.52; *p* < 0.01).

*HBSC*. We found a significant reduction in the number of cigarettes smoked (mean-ranks difference = −4.75, *p* < 0.01).

### 3.3. Middle School Pre-Post Test: Differences

*APEP/G and APEN/G*. Improvements were found, but were not statistically significant (APEP/G: difference = 3.59; *p* < 0.01. APEN/G: difference = 3.77; *p* < 0.2).

*Idea Inventory Questionnaire*. We found a statistically significant improvement in the scale's overall score (difference = 2.86; *p* < 0.01).

*LAQ*. We found significant improvements in the overall score (difference = 15; *p* < 0.001), in the* goal definition* (mean-ranks difference = 10.5; *p* < 0.01), in* expressing positive feelings* (difference = 4.5; *p* < 0.01), in* making requests *(difference = 8; *p* < 0.01), in* expressing unpleasant feelings* (difference = 8.87; *p* < 0.01), in* active listening* (difference = 9.62; *p* < 0.01), and in the* problem-solving* (difference = 4.27; *p* < 0.01).

*HBSC*. We found a significant improvement in the subjective quality of life (difference = 6.8, *p* < 0.01).

### 3.4. High School versus Middle School (Table 3)

*APEP/G and APEN/G*. No differences were found between the groups before (APEP/G: difference = 4.79; *p* < 0.5. APEN/G: difference = 1.79; *p* < 0.8) or at conclusion of the study (APEP/G: difference = 6.78.; *p* = 0.4. APEN/G: difference = 1.79; *p* = 0.8).

*Idea Inventory Questionnaire*. At the end of the implementation, we found a significant difference in favour of the middle school students (difference = 14.45; *p* = 0.05).

*LAQ*. At the end, in favour of the middle school classes we found significant differences in the overall score (difference = 28.14; *p* < 0.01), in* expressing positive feelings* (difference = 16.29; *p* < 0.01), in* making requests* (difference = 30; *p* < 0.01), in* expressing unpleasant feelings* (difference = 20; *p* < 0.01), in* active listening* (difference = 19.04; *p* < 0.01), and in* problem-solving* (difference = 17.7; *p* < 0.01).

*HBSC*. At the end of the implementation we found a significant improvement of* subjective quality of life* (difference = 13.38; *p* = 0.05) in favour of the middle school.

## 4. Discussion

This study, like the previous ones conducted by the same team [5, 21, 29], confirms that school is an important setting for the promotion of mental health [30] and that these initiatives can be successful if (a) they are structured and evidence-based [31]; (b) they are directed towards changing behavioural aspects related to specific objectives that take into account the everyday life of the student in relation to the family environment and the surrounding community [10]; (c) they encourage greater sharing among students of both aspects of the curricular goals and personal/interpersonal growth [32, 33].

In middle as well as in high school students, we observed improvements in their communication skills, goal definition, and self-efficacy in regulating negative emotions, although the latter did not reach statistical significance among middle school students. This research gives us more precise direction regarding the possibility of improvements in the developmental stage. In fact the same approach has been used in high school and middle school where the method was accepted similarly with satisfaction by students and teachers and has been shown, for many of the facets investigated, to be effective as hypothesized by the authors of the handbook [34].

It should be noted that the approach was effective in terms of knowledge of skills (such as defining goals, effectively communicating, and using problem-solving) among students aged 12–14 years of age as well as students aged between 15 and 17 years of age, with some differences observed between the different stages of development. In fact, we observed an increase in functional beliefs and subjective quality of life only in favour of middle school students.

On the other hand, we observed an increase in favour of high school students as regards self-efficacy and healthy behaviours (i.e., decreased number of cigarettes smoked), in consonance with other similar studies [35].

In the area of management of emotions, the absence of improvement in perceived self-efficacy among middle school students might have a dual explanation based on the theories of Bandura [36]. This author argues that the explanatory variables of self-efficacy are numerous and many of them are influenced by the modelling experiences and physiological as well as affective states that suddenly change in the 12- to 14-year age group. Maybe learning self-efficacy skills needs more intensive and continued application and exercise in this group. Additionally, the management of negative emotions requires greater capacity for self-reflection, as argued by the same author, and it is therefore evident that on this scale, a positive impact was not registered in the intervention; in fact a good impact was not registered on the middle school students.

Instead, on the other hand, there is the improvement in changing irrational beliefs. In fact, an improvement in overall score in the middle school students before and after the intervention was registered; this improvement was not observed in the high school students. The statistically significant outcomes in favour of the middle school students could be explained by the greater cognitive flexibility of preteen-aged children. Therefore, it may suppose that emotional education for changing irrational and unrealistic beliefs is more efficient in working with students younger than 15 years of age compared to students aged more than 15 years. This observation agrees with authors who support rational-emotive education courses [37], starting even in primary school and continuing for many years, with a positive effect of this kind of intervention inversely proportional to age [38]. The greater cognitive flexibility of children at younger ages, associated with an attitude of schooling more prevalent in this age group, also explains the better results obtained in learning effective communication and problem-solving skills, at least on a theoretical level. There is no data on the ability to use these skills in everyday life, but it is an undeniable fact that, theoretically, there has been a greater assimilation of this ability. On the other hand, we cannot exclude that these skills are not used in everyday life. In fact, among the skills learned in a significantly better way at younger ages is the communication of pleasant feelings, which is done mostly by middle school students as compared to high school students, as the LAQ questionnaire shows. This result is encouraging because we can hypothesize that with sessions focused on reinforcing these skills at home or in the same school environment; the students could easily get to use them in daily life. In this sense, in subsequent years, a programme of “booster sessions” improving the recognition of emotions, communication, and problem-solving skills would be helpful. In addition, these skills could benefit from the active support of relatives. As a consequence, simple and specific strategies should be developed for the involvement of family members, such as description of previous experience from the students who participated in the programme of the handbook's implementation, the presentation of the results obtained from the effectiveness studies of this implementation, and the presentation of a video illustrating the method and purpose.

The study has a number of limitations. It is not a controlled study; the optimal sample size was not estimated a priori, and the follow-up period should have been extended because the observed improvements might be lost over a longer follow-up period. Moreover, the samples only included students who were resident in a defined catchment area of Southern Italy, which may limit the generalizability of the results and might have introduced some sort of bias, as the students' psychosocial characteristics and the levels of motivation experienced by the teaching local staff may have affected students' participation in and their receptivity and responsiveness to the programme. Therefore, our findings need to be confirmed by further investigations in other areas of Italy. Nevertheless, they are encouraging because they show the effectiveness of even more facets than those previously investigated [21, 29].

The study's limitations are also partially related to the nature of the investigation that has to take into account “practical” factors linked to the feasibility of this type of research that needs to obtain different degrees of consent from the parents, but also the availability of the faculty and the headmaster. These limitations do not allow the results to be generalized, even if they appear in terms of stimulus experimentation in conducting future studies with greater methodological rigour.

## 5. Conclusion

This study suggests that the modified handbook contributed to the improvement of earlier positive results obtained in the other similar studies [23, 29, 34]. Nevertheless, the authors believe that further improvements of the handbook may be provided with a further simplification and a greater emphasis on the exercises.

On the other hand, this study has a number of methodological limitations and it is necessary to replicate it in other southern, central, and northern Italian regions in the future, to examine whether there are differences depending on the geographic location.

It should be noted, however, that there are few methodologically rigorous studies on an international level, rare in Europe, carried out with a controlled randomized methodology and with structured, manualized, and reliable interventions [39]. In fact, in the face of numerous articles on the subject, as reported in the widest and most recent survey [3], only 14 studies fulfilled the research quality criteria, and, among these, only 8 of them received a strong quality rating [39]. Our study also showed that the handbook is applicable in a similar way to both middle school and high school, with interesting differences related to the different facets we investigated. The authors believe that one year after the handbook's implementation, the possible addition of booster sessions will consolidate and verify the degree of acquisition and maintenance of skills.

## Figures and Tables

**Table 1:** Work-units of the handbooks.

	High school, original version		Middle and high school, modified version
(1)	Introduction: purposes, limits and structure	(1)	Introduction: purposes, limits and structure
(2)	How to give constructive feed-back; the importance of distinguishing judgments concerning behaviours/persons, desires/needs and different degrees of emotions	(2)	How to give constructive feed-back; the importance of distinguishing judgments concerning behaviours/persons, desires/needs and different degrees of emotions
(3)	Defining enjoyable personal goals	(3)	Defining enjoyable personal goals
(4)	Structured problem-solving for practical problems	(4)	A SMART goal 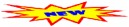
(5)	Expressing unpleasant feelings and active listening	(5)	Expressing positive feelings
(6)	Expressing positive feelings and making requests	(6)	Making requests in a positive way
(7)	Assertive communication training	(7)	Expressing unpleasant feelings
(8)	Increasing the social network	(8)	Active listening
(9)	Conflicts and negotiation	(9)	Events, emotions and thoughts: the Cognitive-Behaviour-Emotion Model 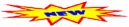
(10)	Improving self-discipline	(10)	Functional/dysfunctional thoughts: the mental virus
(11)	Managing one's own impulses and anger	(11)	Worry-anxiety feeling 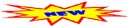
(12)	Self-acceptance	(12)	Put yourself in others' shoes 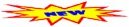
(13)	Functional/dysfunctional thoughts	(13)	Self-acceptance
(14)	Stress management	(14)	Assertive communication training
(15)	Information about mental disorders	(15)	Improving self-discipline
(16)	Main characteristics of depression	(16)	Communicating anger 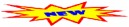
(17)	Maintaining progress	(17)	Controlling one's own aggressively impulses and anger
		(18)	Structured problem-solving for practical problems
		(19)	Maintaining progress

**Table 2:** High and middle school pre-post differences.

Variables	Ranks average Before (Pre)	Ranks average After (Post)	Test	*p*
			Wilcoxon (difference)	
*High-School Group*				
APEP/G (positive Emotions)	30.87	31.17	0.30	0.33
APEN/G (negative Emotions)	32.96	40.26	7.30	**0.01**
APEP/G + APEN/G	30.78	40.36	9.58	**0.02**
Goal definition	16.17	22.95	6.78	**0.01**
Expressing positive feelings	9.50	16.70	7.20	**0.01**
Making request	8.50	11.36	2.86	**0.05**
Expressing unpleasant feelings	17.32	24.84	7.52	**0.01**
Active listening	22.74	26,03	3.29	0.06
Problem-solving skill	10.05	11.86	1.81	0.25
Idea inventory	58.23	52.78	−5.45	0,53

*Middle-School Group*				
APEP/G (positive Emotions)	12.77	16.36	3.59	0.09
APEN/G (negative Emotions)	13.81	17.58	3.77	0.18
APEP/G + APEN/G	13.10	17.46	4.36	0.07
Goal definition	11.00	21.50	10.50	**0.01**
Expressing positive feelings	10.50	15.00	4.50	**0.01**
Making request	8.00	16.00	8.00	**0.01**
Expressing unpleasant feelings	14.00	22.87	8.87	**0.01**
Active listening	17.63	27.25	9.62	**0.01**
Problem-solving skill	14.50	18.77	4.27	**0.01**
Idea inventory	54.02	67.23	13.21	**0.01**

**Table 3:** High school versus middle school differences at the end of the study.

Variables	High school (ranks average)	Middle school (ranks average)	Mann–Whitney (difference)	*p*
APEP/G + APEN/G	55.27	61.20	5.93	0.38
Goal definition	54.56	62.91	8.35	0.16
Expressing positive feelings	52.24	68.53	16.29	**0.01**
Making request	48.27	78.27	30.00	**0.01**
Expressing unpleasant feelings	51.16	71.15	20.00	**0.01**
Active listening	51.44	70.48	19.04	**0.01**
Problem-solving skill	51.84	69.52	17.68	**0.01**
Global LAQ score	48.78	76.92	28.14	**0.01**
Idea inventory	52.78	67.23	14.45	**0.05**
